# University of Pennsylvania

**Published:** 1855-06

**Authors:** 


### UNIVERSITY OF PENNSYLVANIA—PROF. SMITH.

Our readers have doubtless learned before this that Dr. Gibson has resigned the professorship of Surgery which he has occupied for many years in the University of Pennsylvania, and that Dr. Henry H. Smith, of Philadelphia, has been elected his successor. It is not the object of the present notice to discuss the merits of this appointment. We can only say that Professor Smith is favorably spoken of by those who know him, and has been regarded for some years as one of the prominent young surgeons of his native city, and we know of no reason why he should not attain to eminence in the great field upon which he has now entered. It is the manner in which this election has been made that we wish particularly to notice.

Our readers are aware that the appointing power in Medical Colleges (with very few exceptions) resides in a Board of Trustees entirely distinct from the faculty, and not necessarily in the slightest degree under its influence. But as far as we are conversant with the policy of medical schools, the trustees are accustomed to depend mainly upon the advice and recommendation of the Professors in filling vacancies whenever they occur, and we have never *known* an instance or heard of one until the recent appointment at Philadelphia, where the recommendation of the faculty has been disregarded. In the present case it is notorious that their opinions were of no weight with the majority of the board of trustees, and that Professor Smith was elected to the vacant chair not only without the concurrence of the Professors, but against their expressed wishes. The resignation of Professor Gibson had been anticipated for some time, and his colleagues had naturally been earnestly occupied in looking for a suitable successor, and it appears that they were unanimous in recommending a distinguished surgeon and teacher residing in a distant State*.

Of the reasons which produced this strange anomaly we are not informed. It may be that state or city pride required the election of a Philadelphian, one "native and to the manner born." Such a motive has been suggested as likely to be operative in no small degree; if it was so, we must be allowed to say that it was an exceedingly narrow and illiberal one, unworthy the governors of an ancient and honorable institution, for it should be remembered that a great school of medicine is not merely an institution of a single state, but extends its influence to all parts of the Union. The medical profession of the whole country has a deep interest in every prominent medical college, and unless that interest is acknowledged and consulted in the election of the most competent teachers who can be obtained, irrespective of family influence or without regard to what State or city may chance to have been their home, the neglect will be speedily felt in diminished patronage and waning prosperity. We insist that it is the duty of the governors of all those schools, whose prominence and prosperity enable them to select the incoming members of their Faculty, to be governed in their choice solely by the ability of the candidate to perform in the highest degree the duties of his Professorship. This has always been the policy of the University of

* We are informed that the candidate recommended by the faculty received the earnest support of all its members except one, who preferred to keep aloof from the whole matter.

Louisville, and we can but think that it has essentially contributed to its prosperity.

If the city in which a medical school is established can furnish a candidate for a vacant Professorship equal in all respects to one who lives at a distance, it would certainly be proper and expedient to elect him, otherwise the claim of residence should be disregarded.

We do not intend by these remarks to cast any disrespect upon Professor SMITH. He may be, for any thing we know to the contrary, the most competent man who could have been obtained in the whole country, but it seems that those who are now his colleagues thought otherwise.

It is certainly no disparagement to the character of the medical profession of any city, even of Philadelphia, so justly distinguished for the eminence of her physicians, to find it advisable to look abroad for the occupant of an important medical professorship. It is not always an easy matter to find that rare combination of excellencies so essential in constituting a great teacher, and it may chance now and then that some lesser town has developed and matured the ability and capacity required for the occasion.

Extensive professional learning, profound attainments in the special department to be taught, together with consummate ability as a practitioner, are not the only qualifications necessary for the occupant of a Professor's chair. To these he must add the ability to teach, and to become eminent, he must possess that faculty in an extraordinary degree. Men of highly respectable attainments are to be found in numbers gratifying to our national pride in all our cities, but the combination of qualities just referred to is much less frequent.

But the point connected with the late action of the trustees of the University of Pennsylvania which more especially arrested our attention and led to the writing of this article, is the constant report, that in the election of Professor SMITH the trustees disregarded what may properly be called the unanimous recommendation of the Faculty. This is certainly an unusual procedure, and it seems to us one, if general, certain to produce the most disastrous results. The prosperity and usefulness of every medical college is largely dependent upon the character and qualifications of its Professors. Splendid buildings, collossal museums, extensive libraries and apparatus, together with illimitable hospital facilities and anatomical supplies, will not make a great medical school, unless these means are in the hands of an accomplished and efficient Faculty. This is felt and admitted by all who are experienced in the history and management of such institutions, and consequently whenever a vacancy occurs in the board of teachers, the utmost anxiety exists in the minds of the remaining members until it is satisfactorily filled. If the question is seriously asked—which of the two bodies constituting the officers of a medical college, Trustees or Professors, is best qualified to judge of the qualifications and decide upon the merits of a candidate for a vacant chair, it seems to us there can be no doubt as to the answer. The members of the Faculty are directly and personally interested in every thing pertaining to the school with which they are connected. Its interests and reputation are identical with their own, and they cannot possibly be governed by any motives, in their choice of a new Professor, which do not tend directly to its present and future prosperity. Not only their income, but what with men of generous minds is of greater importance, their professional reputation and character are largely dependent upon the reputation and character of their colleagues, and hence the fidelity of the Professors of a medical college to its true interests may always be relied upon. If they commit error it will always be the result of a mistake in judgment, while their position renders them of all other men best qualified to judge in this matter, and experience has abundantly proved that they are rarely mistaken. The vigorous but usually generous competition which characterizes medical teaching in this country, renders those engaged in it vigilant in marking the development and progress of those who are attaining positions of distinction and influence, and the desire not to be distanced in the race by rival schools, causes the members of the faculty of all our leading institutions to be exceedingly circumspect in their selection of new colleagues so as to add to their strength and prosperity. And what other body of men, we ask, can possess the same means of knowing, and consequently of judging of the varied qualifications requisite for a medical professorship, or of the respective merits of rival candidates? The

Boards of Trustees are usually constituted from the various professions and avocations of life with but a small sprinkling of physicians. Judges and lawyers, statesmen and divines, merchants and bankers, are liberally arrayed upon the lists, and however eminent they may be in their respective spheres, and however anxious they may be for the prosperity of the college under their charge, they must of necessity be poorly qualified to judge in this important matter. Their knowledge must come from others, and if they prefer to disregard the opinions of those most deeply interested, their own professors, and trust to letters of recommendation, certificates of qualification, or even their own personal acquaintance, there will be a strong liability, amounting often to a certainty, that they will be led into the commission of serious errors. State or city pride, family influences, exerted in behalf of a relative who chances to be an aspirant, personal friendships, or even enmities, professional jealousies and rivalries, all may be brought to bear upon men whose occupations and studies have not fitted them for this delicate duty, and whose more indirect and consequently less absorbing interest renders them less vigilant. Physicians are the best judges of the professional character and qualifications of their brethren, and medical teachers know best what medical teachers are and what they should be.

It seems to us that there is but one proper course to be pursued in a matter so important as the election of a medical professor, one which is so intimately connected with the prosperity of all our medical schools. It seems to us that in all cases where the faculty are unanimous in the recommendation of a candidate for a vacant chair, the trustees should unhesitatingly approve and confirm it, and that only where disagreements exist among the professors should the discretion of the trustees be exercised in the election, and we are confident that where this custom has prevailed, and we believe it to be almost universal, the trustees have rarely if ever had occasion to doubt the wisdom of their action.

